# Globalization of clinical trials in oncology: a worldwide quantitative analysis

**DOI:** 10.1016/j.esmoop.2024.104086

**Published:** 2024-12-18

**Authors:** F. Izarn, J. Henry, S. Besle, I. Ray-Coquard, J.-Y. Blay, B. Allignet

**Affiliations:** 1 Department of Medical Oncology, Centre Léon Bérard, Lyon, France; 2 Department of Human and Social Sciences, Triangle, UMR 5206, ENS de Lyon, Lyon, France; 3 Human and Social Sciences Department, Centre Léon Bérard, Lyon, France; 4 Université Claude Bernard Lyon 1, INSERM 1052, CNRS 5286, Centre Léon Bérard, Centre de Recherche en Cancérologie de Lyon, Institut Convergence PLAsCAN, Lyon, France; 5 Université Claude Bernard Lyon 1, Lyon, France; 6 Department of Radiation Oncology, Centre Léon Bérard, Lyon, France; 7 Univ Lyon, INSA-Lyon, Université Claude Bernard Lyon 1, CNRS, Inserm, CREATIS UMR 5220, U1294, Lyon, France

**Keywords:** clinical trials, oncology, geography, inequality, ethical concerns

## Abstract

**Background:**

Over the past two decades, the globalization of oncology clinical trials has expanded, yet significant disparities persist across countries. This study aimed to evaluate these geographical inequalities, the evolution of trial phases, and the adherence to ethical standards according to the World Bank’s income group.

**Materials and methods:**

The ClinicalTrials.gov database was searched and recorded in June 2024. We analyzed data from 87 748 oncology clinical trials conducted between 2000 and 2021, across high-income (HICs), upper-middle-income (UMICs), lower-middle-income (LMICs), and low-income countries. Key metrics included trial density, funding sources, registration timing, and trial phase distribution.

**Results:**

The number of oncology trials increased significantly, with a mean absolute annual rise of 266.6 trials, with China currently being the leading site of early- and validation-phase trials. While HICs still present the highest trial densities, UMICs showed a notable increase in early-phase trials, reflecting a shift in research dynamics. However, despite these advances, 76.4% of countries still had no new trials initiated by 2024. Additionally, ethical practices saw improvement from 2005 to 2021 with an increase in pre-commencement registration (from 9.2% to 58%, *P* < 0.0001), and more validation-phase trials with a survival variable as the primary outcome (from 40% to 59.6%, *P* < 0.0001).

**Conclusions:**

Despite the growth in oncology clinical trials, significant disparities in trial distribution and access remain, especially in LMICs. Continued investments in research infrastructure and adherence to ethical standards are crucial to ensure that clinical research benefits are equitably distributed, particularly in regions with the greatest need for advanced cancer therapies.

## Highlights

• A significant rise in oncology research was observed, from 638 registered trials starting in 2000 to 6571 in 2021.
• 76.4% of countries had no new oncology trials by 2024.
• Most early- and validation-phase trials now start in China.
• Ethical and integrity practices improved, with pre-enrollment registration rising from 9.2% in 2005 to 58% in 2021.
• Survival as the primary outcome in validation trials increased from 40% to 59.6%.

## Introduction

Clinical research in oncology is essential for advancing medications, medical devices, and overall medical practices. It provides early access to innovative therapies that have the potential to be highly effective and ensures evidence-based management. Access to clinical trials and new treatments is increasingly sought after by health care professionals, public authorities, and, for several years now, by patients themselves.^1^, ^2^, ^3^ However, this access remains subject to geographical disparities.^4^ During the last 20 years, a sharp increase in the number of clinical trials was observed, particularly in developing countries.^5^^,^^6^ The worldwide population representativity in clinical trials is essential to produce generalizable results but urgently remains to be improved.^7^

The growing involvement of upper-middle-income and lower-middle-income countries (UMICs and LMICs, respectively) raises concerns. Participants and clinical research structures of low-resource countries may be exploited due to fewer legal and ethical restrictions, and due to lack of access to health care for financial reasons. Institutional review boards (IRBs) are intended to enforce ethical standards and prevent such abuse by ensuring that research adheres to strict ethical guidelines.^8^ Although access to post-trial therapies is a fundamental principle outlined by the Council for International Organizations of Medical Sciences (CIOMS) guidelines,^9^ populations in these countries often do not have access to these new treatments, even when they have been developed and tested locally.^10^ Additionally, the provision of compensation and ancillary care during trials, although well intentioned, can raise ethical questions about potential undue inducement for participation.

In this context of globalization, transparency and adherence to ethical standards are crucial.^11^ However, while some quantitative data on clinical trials have been reported, evaluations of their quality remain limited, particularly in lower-resource settings.^11^

Trial registration is required by the International Committee of Medical Journal Editors (ICMJE) and World Health Organization since 2005 and 2006, respectively. The most widely used and most comprehensive open-access trial registry is ClinicalTrials.gov, which was created in 1999 and hosted by the United States National Institutes of Health (NIH). Using this reference database, we aimed to provide a comprehensive overview of clinical research in oncology worldwide in 2024, and its qualitative and quantitative evolution during the last two decades.

## Materials and Methods

### Data sources and study selection

We searched ClinicalTrials.gov (https://clinicaltrials.gov/) on 13 June 2024, for all trials related to oncology, using the string ‘cancer’ and its 26 related synonyms (Supplementary Table S1, available at https://doi.org/10.1016/j.esmoop.2024.104086), without other initial restriction. Since ClinicalTrials.gov was created only in 1999, we excluded trials with a start date before 1 January 2000, or with an unavailable start date. We focused on the period from 2005 to 2021, as trial registration has been required by the ICMJE since 2005, and 2021 represents the most recent year with a comprehensive record of oncology trials due to delays in protocol acceptance and publication on the ClinicalTrials.gov platform.

Since trials’ information reported in ClinicalTrials.gov is not restricted to ontologies, we further refined the extraction conserving only trials that included text pattern related to oncology, tumor histology, molecular data, hematological malignancies, and cancer treatments in the study title, condition/disease, and/or intervention (details in Supplementary Table S1, available at https://doi.org/10.1016/j.esmoop.2024.104086). This pattern recognition was carried out using the package stringr version 1.5.0 on R software version 4.4.1 (R Foundation for Statistical Computing, Vienna, Austria).

Countries were divided into three income groups (low, middle, and high) according to the World Bank’s classification (https://www.worldbank.org/), based on gross national income per capita. The population of each country was obtained from the world population prospects 2022 of the Department of Economic and Social Affairs of the United Nations (https://population.un.org/wpp/). Population and trial sites of French, British, and Dutch overseas territories were combined with the data of France, UK, and the Netherlands, respectively.

### Variables of interest

In addition to the absolute number of trials, other metrics were evaluated to better consider the research activity of each country. The number of trial sites was calculated by multiplying the number of trials by the number of sites, to consider the multicenter design. The number of trial site-years was the product of the trial sites and the duration of the trials.^12^ As an example, a trial (*n* = 1) that started in 2015 and was completed in 2016 (*n* = 2 calendar years), involving three recruiting sites in the USA (trial sites, *n* = 3), would generate 6 trial site-years. Not to underestimate the activity of small countries, the trial site density was obtained by dividing the trial sites per year by the country population in millions.

For each income group defined by the World Bank, we assessed the absolute number of trials, trial sites, trial site-years, and, for each trial, the multicenter design, number of enrolled patients, interventional or observational status, study phase, masking, and funding source (industry versus other). In case of a trial with recruiting sites in various income groups, the trial was weighted proportionally to the number of sites in each group (i.e. if 2 recruiting sites in Canada and 1 in Mali, the trial will be accounted as 2/3 and 1/3 trials in high- and low-income groups). The study phase was divided into three categories: early phases (including early phase I and phases I, I-II, and II), validation phases (phases II-III and III), or other.

To carry out a preliminary evaluation of the clinical pertinence and ethics of the trials according to the income group, we evaluated the proportion of validation-phase trials with a survival variable as the primary outcome and the proportion of trials that were registered before the study start.

### Statistical analyses

Categorical variables were presented as count (percentages) and compared using chi-square tests. Continuous variables were presented as median [interquartile range (IQR)] and compared using Student’s *t*-tests or one-way analysis of variance according to the number of groups. To assess the increase in the number of starting trials between 2000 and 2021, linear Pearson correlation was carried out. Since trial registration is a long process and some trials are registered after the start date, we excluded the subsequent years so as not to underevaluate this linear correlation. To ensure the robustness and interpretability of the results, cases with missing data were excluded for analyses involving missing variables, with no imputation carried out. Tests were carried out on relevant data, excluding observational trials when analyzing allocation, masking, and phases. A two-sided *P* value <0.05 was considered significant. Statistical analyses and figures were carried out using R software version 4.4.1 (R Foundation for Statistical Computing).

## Results

### Panorama of the clinical research in oncology in 2024

**Figure 1:**
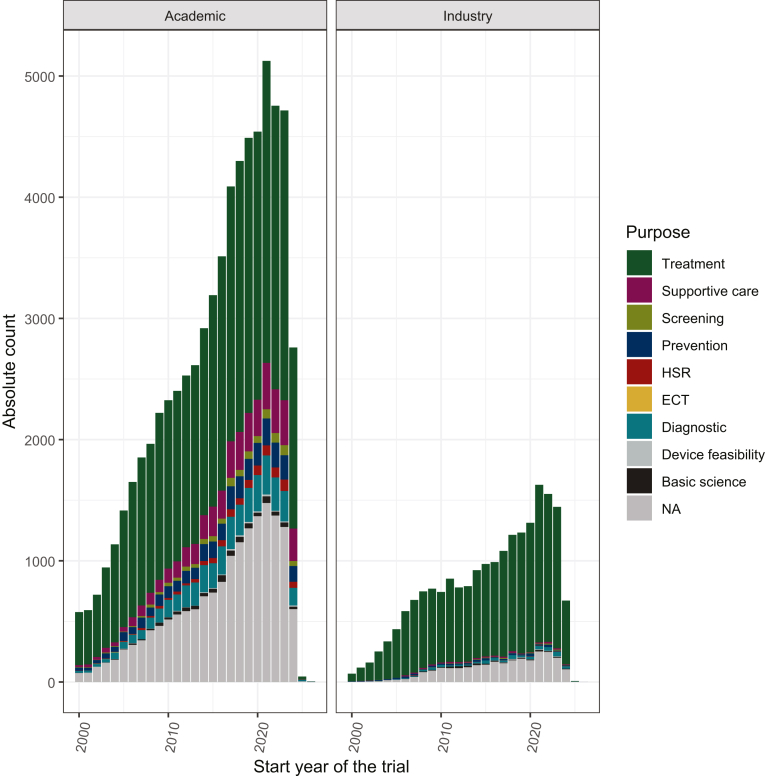
Number of starting trials each year, according to their funding and purpose. ECT, educational/counseling/training; HSR, health services research; NA, not available.

The inclusion criteria were met by 87 748 trials (Supplementary Figure S1, available at https://doi.org/10.1016/j.esmoop.2024.104086), with 29 340 ongoing trials. Most of them were monocentric (*n* = 17 756, 60.5%), interventional (*n* = 23 307, 79.4%), with a treatment purpose (*n* = 18 111, 61.7%), and with a non-industrial funding (*n* = 23 304, 79.4%) (Table 1). A large increase in oncological research was observed, with 266.6 (95% confidence interval 249.4-283.8) additional trials per year between 2000 and 2021 (638 starting trials in 2000 versus 6571 in 2021; *r* = 0.99, *P* < 0.0001) (Figure 1, Supplementary Figures S2 and S3, available at https://doi.org/10.1016/j.esmoop.2024.104086). Regarding quality and ethics, 39.4% of trials were still registered after the first patient inclusion, and 69.8% and 31.5% of validation phases were open and had a non-survival primary outcome, respectively.

**Table 1:** Characteristics of worldwide ongoing trials

	Ongoing trials (*N* = 29 340)
Study type, *n* (%)	
Interventional	23 307 (79.4)
Observational	6033 (20.6)
Multicenter design, *n* (%)	
No	17 756 (60.5)
Yes	11 584 (39.5)
Funder type, *n* (%)	
Industry	6036 (20.6)
Academic or other	23 304 (79.4)
Enrollment, median (range)	87 (38-240)
Allocation, *n* (%)	
Randomized	8464 (28.8)
Non-randomized	4058 (13.8)
Missing	10 785 (36.8)
Non-relevant^a^	6033 (20.6)
Masking, *n* (%)	
None	20 386 (69.5)
Single	1083 (3.7)
Double	777 (2.6)
Triple	433 (1.5)
Quadruple	603 (2.2)
Missing	6 (0.0)
Non-relevant^a^	6033 (20.6)
Phases,^b^*n* (%)	
Early phase	13 997 (47.7)
Validation phase	2603 (8.9)
Pharmacovigilance	377 (1.3)
Missing	6330 (21.6)
Non-relevant^a^	6033 (20.6)
Purpose, *n* (%)	
Treatment	17 004 (62)
Supportive care	1406 (5.1)
Diagnostic	1270 (4.6)
Prevention	867 (3.2)
Screening	316 (1.2)
Health services research	300 (1.1)
Basic science	192 (0.7)
Device feasibility	53 (0.2)
Missing	6009 (21.9)
Correct registration, *n* (%)	
Yes	15 992 (58.3)
No	9624 (35.1)
Missing	1801 (6.6)

a Observational trials.

b Early phase: early phase I, phases I, I-II, and II. Validation phase: phases II-III and III.

When focusing on trials with treatment purposes (*n* = 18 111), most are monocentric (*n* = 9622, 53.1%), have non-industrial funding (*n* = 13 006, 71.8%), are open (*n* = 16 374, 90.4%), and enroll a median of 61 patients (IQR 30-153 patients).

### Clinical research according to income group

**Figure 2:**
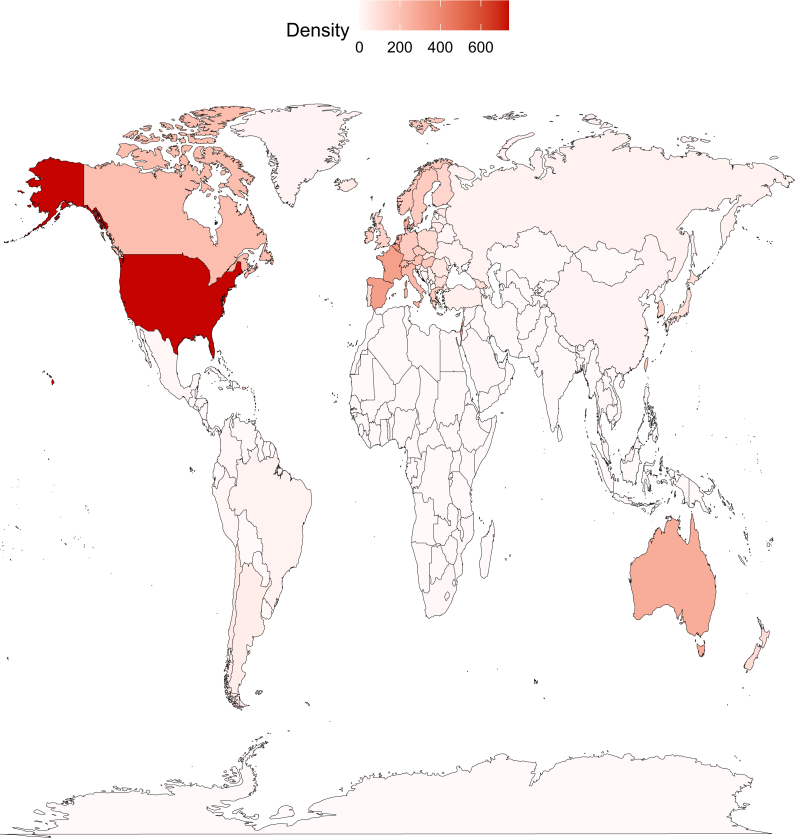
Ongoing trials’ density map (per million of habitants).

High-income countries (HICs), UMICs, LMICs, and low-income countries (LICs) have 1066.4 (0-1157.9), 459.9 (186-675.2), 24.7 (0-32.9), and 1 (1-1) ongoing trial sites, respectively (*P* < 0.0001) (Figure 2). As expected, income group is correlated with the continent of the countries (*P* = 0.0005). As examples, all 3 Northern American and 45 of 46 (97.8%) European countries are HICs or UMICs, whereas 46 of 54 (85.2%) African countries are LMICs or LICs. While 162 (76.4%) countries still do not have any clinical trials starting in 2024, 38 of the 40 countries with the highest trial density are HICs (Table 2 and Supplementary Table S2, available at https://doi.org/10.1016/j.esmoop.2024.104086). The proportion of trials funded by industry varied from 5.6% in LICs to 44.6% in LMICs (*P* < 0.0001), with important variations even in HICs (Supplementary Figure S3, available at https://doi.org/10.1016/j.esmoop.2024.104086). Among multicenter trial sites, 96.2% (*n* = 84 415) involved countries from only one income group, primarily from HICs (*n* = 67 196, 79.6%) or UMICs (*n* = 15 626, 18.5%).

**Table 2:** Characteristics of clinical trials since 2000 according to the World Bank income group

Characteristics	High income	Low income	Lower middle income	Upper middle income	*P* value
Enrollment, median (range)	53.5 (22.84-149.78)	185.5 (47.43-1018.59)	79.5 (49.17-167.83)	79.5 (35.85-201.59)	0.9
Multicenter design, *n* (%)					<0.0001
Yes	29 975.1 (43.1)	15.4 (26.8)	395.5 (23.4)	3450.8 (21.1)	
No	39 634 (56.9)	42 (73.2)	1295 (76.6)	12 920 (78.9)	
Study type, *n* (%)					
Interventional	56 176.1 (80.7)	37.1 (64.7)	1211.3 (71.7)	13 239.1 (80.9)	
Observational	13 419.8 (19.3)	20.2 (35.3)	479.1 (28.3)	3130 (19.1)	
Missing	13.2 (0)	0 (0)	0.1 (0)	1.7 (0)	
Study status,^a^*n* (%)					<0.0001
Completed	32 491.4 (46.7)	29 (50.6)	803.8 (47.5)	3739.2 (22.8)	
Ongoing	21 661.8 (31.1)	19 (33.1)	444 (26.3)	7212.1 (44.1)	
Stopped early	9656.1 (13.9)	4 (7)	63 (3.7)	580 (3.5)	
Unknown or others	5799.8 (8.3)	5.3 (9.3)	379.7 (22.5)	4839.4 (29.6)	
Trial phase,^b^*n* (%)					<0.0001
Validation phase	5269.5 (7.6)	4.7 (8.2)	254.6 (15.1)	2097.5 (12.8)	
Early phase	35 202.9 (50.6)	5 (8.7)	313.1 (18.5)	7637.4 (46.7)	
Pharmacovigilance	1146.6 (1.6)	1 (1.7)	99.4 (5.9)	495.6 (3)	
Missing	14 570.4 (20.9)	26.4 (46.1)	544.4 (32.2)	3010.4 (18.4)	
Non-relevant	13 419.8 (19.3)	20.2 (35.3)	479.1 (28.3)	3130 (19.1)	
Masking, *n* (%)					<0.0001
None	48 176.7 (69.2)	26.4 (46.1)	690.7 (40.9)	10 845.4 (66.2)	
Single	2585.3 (3.7)	7.3 (12.8)	192.9 (11.4)	910.6 (5.6)	
Double	2168.3 (3.1)	1.3 (2.3)	190 (11.2)	590.7 (3.6)	
Triple	1138.8 (1.6)	0 (0)	71.7 (4.2)	320.5 (2)	
Quadruple	1475.6 (2.1)	2 (3.5)	65.6 (3.9)	566.8 (3.5)	
Missing	14 064.4 (20.2)	20.2 (35.3)	479.6 (28.4)	3137 (19.2)	
Non-relevant^a^	13 419.8 (19.3)	20.2 (35.3)	479.1 (28.3)	3130 (19.1)	
Correct registration, *n* (%)					<0.0001
Yes	32 899.7 (47.3)	32.6 (56.8)	581.1 (34.4)	6195.7 (37.8)	
No	36 709.4 (52.7)	24.8 (43.2)	1109.4 (65.6)	10 175.2 (62.2)	
Survival primary outcome,^c^*n* (%)					<0.0001
Yes	2448.1 (48.4)				
No	2614.5 (51.6)				

a Completed: completed. Ongoing: not yet recruiting, recruiting, enrolling by invitation, active not recruiting, suspended. Stopped early: terminated, withdrawn.

b Early phase: early phase I, phases I, I-II, and II. Validation phase: phases II-III and III.

c Among validation-phase trials.

### Trial phase according to income group

**Figure 3:**
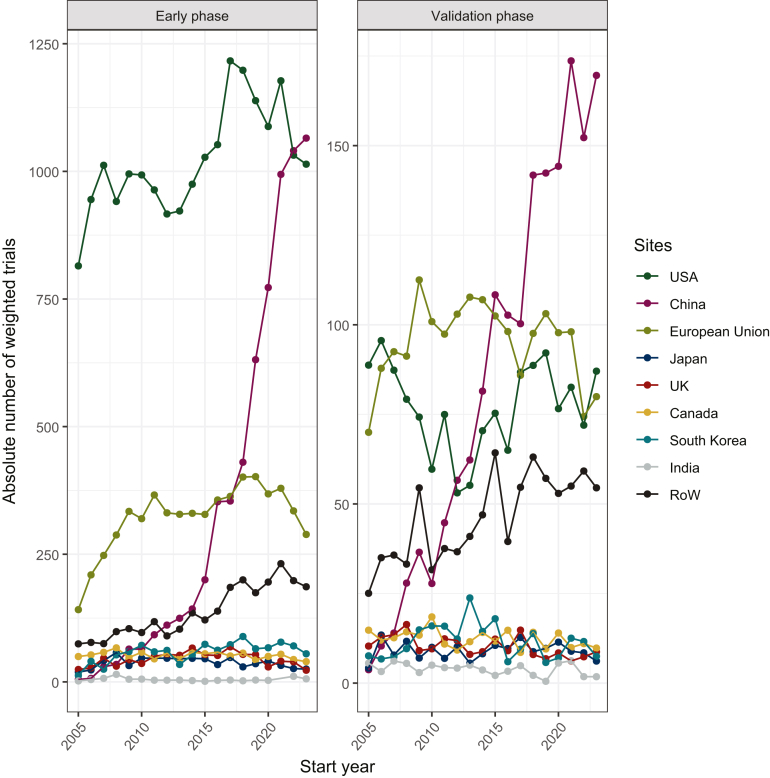
Evolution of the weighted count of early- and validation-phase trials from 2005 to 2023. RoW, rest of the world (including all countries not specified under sites).

Overall, there was a decline in early-phase trial proportion between 2005 and 2021, from 61.7% to 44.2% of all registered trials (*P* < 0.0001). In HICs, this proportion decreased from 62.9% (*n* = 1116.3 trials) in 2005 to 42% (*n* = 1919.2) (*P* < 0.0001) in 2021. On the opposite, UMICs saw an increase from 38.8% (*n* = 22.8) to 51.8% (*n* = 1042.1) (*P* = 0.04). The access to early-phase trials remains highly limited in other countries, even in the lower-middle-income group (*n* = 24 trials in 2021, 14.9%). While most early- and validation-phase trials were in the USA or in the European Union in 2005, China is the leading site of validation-phase trials since 2015 and of early-phase trials since 2023 (Figure 3).

### Clinical pertinence and ethics

An improvement in the timing of study registration was observed worldwide from 2005 to 2021, from 9.2% to 58% (*P* < 0.0001), and was homogeneously observed worldwide (Supplementary Figure S4, available at https://doi.org/10.1016/j.esmoop.2024.104086). Pre-commencement registration of trials rose from 9.4% (*n* = 166.7) to 65.1% (*n* = 2974.9) in HICs, from 4.6% (*n* = 2.7) to 43.4% (*n* = 872.8) in UMICs, and from 3.4% (*n* = 0.6) to 37.4% (*n* = 60.3) in LMICs (*P* < 0.0001) (Supplementary Figure S5, available at https://doi.org/10.1016/j.esmoop.2024.104086). Pre-enrollment registration of trials with treatment purposes drastically increased in this period, from 10.6% to 66.7% (*P* < 0.0001).

The proportion of validation-phase trials with a survival variable as the primary outcome also increased worldwide from 40% to 59.6% (*P* < 0.0001) (Supplementary Figure S6, available at https://doi.org/10.1016/j.esmoop.2024.104086). In HICs and UMICs, it rose from 39% (*n* = 83) to 58.7% (*n* = 138.3) (*P* < 0.0001), and from 49% (*n* = 5.4 trials) to 64.2% (*n* = 126.1 trials) (*P* = 0.21). On the contrary, LMICs saw a decrease from 56.6% (*n* = 3.6) in 2005 to 26.7% (*n* = 5.6) in 2021 (*P* = 0.01).

## Discussion

Over the last 20 years, there has been a sharp increase in the number of cancer clinical trials, meeting a public health need. Indeed, cancer is the second leading cause of death worldwide,^13^ with rising incidence and a growing demand for innovative treatments. Despite an increase in cancer research in most countries worldwide, major disparities remain between subgroups of countries. HICs account for most ongoing trials over the past 20 years, a trend attributable to their larger research infrastructures, administrative human resources, close collaboration between fundamental laboratories and translational/clinical teams, more robust regulatory environments, and greater availability of public and private funding. In contrast, LICs encounter substantial barriers, including underdeveloped research infrastructure, a lack of skilled personnel, less stringent regulatory systems, and insufficient funding.^14^^,^^15^ These challenges have contributed to their slower clinical trial development.

This highlights a significant global cancer funding paradox, where 5% of global resources for cancer are spent in LICs and middle-income countries (MICs), yet these countries bear almost 80% of the disability-adjusted life-years lost to cancer worldwide.^16^ These disparities underscore the urgent need for equitable distribution of research funding and resources to address the global cancer burden effectively.^7^ We report an increase in early-phase trials conducted in UMICs, and that China is now the leading site of early- and validation-phase trials. Our results do not separate delocalization of trials from HICs to UMICs from local increase in research and development teams. The proportion of each is likely to vary as the UMIC category encompasses countries such as Botswana and China.

The inclusion of more diverse population could also translate into better consideration of subtypes that are more prevalent in these regions,^17^ such as nasopharyngeal cancers in Asia, cervical cancers in Africa, or hepatobiliary cancers in India and South-East Asia. This scientific consideration is also an ethical imperative. As there is a growing recognition of the need to include pregnant women and other vulnerable groups in clinical trials, the inclusion of geographically and genetically diverse populations is essential to accurately address specific disease burdens. It is also recognized that certain cancers may exhibit unique characteristics in specific populations, further justifying the need for diverse representation in clinical trials. To date, these diseases whose burden lies mainly in LICs and MICs are investigated seven to eight times less than relevant diseases in HICs.^18^ In addition, development of clinical trial infrastructures can improve quality of care for all patients of the region, independently of their individual study participation. Fostering international cooperation and establishing public–private partnerships (PPPs) can be essential strategies. Such collaborations can provide LICs with the necessary resources, training, and funding to develop sustainable research infrastructure, while also promoting ethical standards and strengthening local expertise.^14^^,^^15^

Part of the increase in the number of cancer clinical trials is indeed driven by the expansion of national and international collaborations, along with the organization of research and treatment initiatives. Many collaborative groups, such as the African Organisation for Research and Training in Cancer (AORTIC), the Asian Cancer Research Group (ACRG), the American Society of Clinical Oncology (ASCO), and the European Society of Medical Oncology (ESMO), have been created. These groups facilitate international networks, promote clinically relevant projects, enhance member expertise, and publish guidelines for up-to-date standards of care. Additionally, international organizations have established accreditations, such as the European comprehensive cancer center excellence label by the Organisation of European Cancer Institutes (OECI), to promote high-quality oncology practice and integrate translational research. Furthermore, grants from ASCO, ESMO, and the European Research Council have significantly facilitated research expansion. Conquer Cancer, the ASCO’s foundation, provided >500 million US dollars in research and education funding through 9200 grants and awards in 89 countries.^19^ Their role in harmonizing practices also contributes to this expansion. As an example, the standardized tumor response assessment using RECIST resulted from a transatlantic cooperation between the European Organisation for Research and Treatment of Cancer (EORTC), the National Cancer Institute of Canada Clinical Trials Group (now Canadian Cancer Trials Group), and the United States National Cancer Institute.^20^

However, despite these advancements, there are still significant challenges to address. Conducting clinical trials on limited ethnic groups poses risks regarding the generalizability of the results. When trials are not representative of the global population, the findings may not accurately reflect the efficacy and safety of treatments across different ethnicities. This lack of diversity may fail to address variations in drug metabolism, genetic factors, and disease prevalence among diverse populations. In addition to non-stringent exclusion criteria, ensuring a diverse participant pool in clinical trials is essential to develop treatments that are effective and safe for all human population, thereby improving the global applicability of research results.^21^ Some solutions may be suggested, such as requiring more demographic characteristics for trial registration by the NIH, requiring information on the representativeness by journal editors and ICMJE, or inclusion of participant representativeness in the score-driving criteria of grant proposal reviews.^22^ On the contrary, the European Medicines Agency (EMA) and Food and Drug Administration (FDA) may require that trial recruitments reflect demographics of the disease to approve new treatments. Fortunately, UMICs such as China are experiencing an increase in clinical trial density, demonstrating growing interest in biomedical research and, in this example, Chinese massive investments in science and technology. Real-world data can evaluate part of this benefit–risk balance in underrepresented populations,^23^ but this evaluation is carried out a posteriori, with potential uncontrolled biases.

Over the past two decades, China has implemented extensive regulatory reforms to accelerate drug approval processes and align with international standards, particularly through initiatives like priority review and conditional approvals, which have notably improved access to innovative oncology treatments.^24^ Through initiatives like the National Clinical Research Centres (NCRC), China has substantially increased its investment in clinical research infrastructure, fostering high-quality, multicentric studies and international collaboration to strengthen its role in global clinical research, especially in priority areas such as oncology.^25^ On the contrary, PPPs have been instrumental in advancing clinical research infrastructure in China, with government reforms encouraging private investment to support the expansion of research facilities and improve access to clinical trials, especially in underserved regions.^26^

In addition to these considerations, it is also worth noting that phase III trials require larger sample sizes, and that lack of recruitment is the most frequent cause of clinical trial discontinuation. The recruitment can be speeded up when trials are conducted in these densely populated countries, in which there are often larger treatment-naïve populations with a higher incidence of more advanced disease.^13^ However, it is crucial to ensure that this recruitment is scientifically justified and benefits the populations involved, rather than being driven by convenience or economic reasons. In addition, while the cost per participant may be lower in MICs compared with HICs, it is essential to avoid viewing these factors as mere advantages without considering the ethical implications. An important issue arises regarding access to therapeutics when they are developed in MICs. Only 9% of MICs that participated in trials leading to FDA approval had access to these drugs 5 years after approval, compared with 46% of HICs.^10^ These data raise concerns about the risks of research parasitism experienced by MICs, who do not always seem to benefit from advances in research even though it is carried out in their own countries. This issue is further complicated by the very high costs of many new oncology drugs, which can be prohibitive for health systems in MICs.

Nevertheless, it is encouraging to note that our study highlights improvements in the timing of study registration. Pre-commencement registration was recommended by ICMJE since 2005 and became mandatory in the Food and Drug Administration Amendments Act of 2007. From 2005 to 2021, there was a significant increase in the proportion of trials registered before their start, especially in HICs and UMICs. This pre-commencement registration is crucial to ensure transparency, quality, and accountability in clinical research. This improvement reflects a growing adherence to international standards and best practices. This progress has been notably supported by regulatory agencies such as the FDA and EMA. These agencies not only facilitate the development of clinical trials but also work to align their procedures, which simplifies the approval process for new therapies across different regions. However, it is important to approach these developments with caution. While regulatory alignment and conditional approvals can expedite the availability of new treatments, they also carry the risk of being influenced by the pharmaceutical industry, potentially leading to a relaxation of standards that could compromise patient safety.

Additionally, the increase in the proportion of validation-phase trials with survival as the primary outcome, particularly in HICs and UMICs, translates into an improvement in the clinical relevance of these trials. However, this progress is not uniform worldwide, as a decline in the proportion of robust primary outcomes was observed in LMICs. Wells et al.^17^ reported complementary data and biases: surrogate endpoints are frequently used in breast cancer trials, substantial benefits are more likely in lung cancer trials, and gastrointestinal cancer trials tend to be published in lower-impact journals. Moreover, an analysis of 307 randomized controlled trials conducted in China and published in 2004 found that 90% of these trials did not report research ethics board review of the protocol.^27^ These findings underscore the need for ethical oversight and equitable practices in global clinical research collaborations to ensure the reliability and scientific integrity of trial outcomes.

To implement these ethical principles in practice, standards and guidelines have been established. Firstly, IRBs and international standards, such as the CIOMS guidelines, play a crucial role in upholding ethical standards across diverse trial settings. These guidelines ensure fair recruitment practices, accessible informed consent, and strict ethical norms, even for underrepresented groups. Secondly, Good Clinical Practice (GCP) guidelines developed by the International Council for Harmonisation (ICH) provide a global framework for maintaining rigorous standards throughout all phases of clinical trials. Adherence to GCP has promoted transparency and accountability while enhancing international cooperation, facilitating multicentric trials, and safeguarding participant rights.

It is important to recognize that the focus of cancer research has evolved over the past decades. The important biological and technological innovations allowed development of personalized treatments considering biological and molecular data. An important issue of precision oncology is the increase in treatment cost. The production of biosimilars by MICs and LICs such as icotinib, a Chinese epidermal growth factor receptor inhibitor,^28^ could possibly improve universal patient access to effective affordable treatments.^29^ However challenges remain, including concerns over immunogenicity, interchangeability, extrapolation of indications, and measures to prevent substandard products and to maintain cold-chain, and variable regulatory standards can limit their adoption.^30^^,^^31^ On the contrary, better comprehension of cancer biology and development allowed apparition of pharmaceutical supportive care, screening, and prevention trials. Furthermore, many patient associations were created that had a role in clinical research orientation and probably partly motivated the expansion of non-pharmaceutical trials.^32^

Given the disparities and challenges highlighted in this discussion, certain areas require ongoing focus and investment. Strengthening research infrastructure and capacity-building in LMICs remains crucial. This will not only enhance the ability of these regions to conduct high-quality clinical trials but also ensure that the populations most affected by specific diseases are adequately represented in global research efforts.^33^ Continued support in these areas is essential for reducing health disparities and fostering equitable access to innovative treatments worldwide. To this end, the European & Developing Countries Clinical Trials Partnership (EDCTP) is ongoing for 20 years. This public–public partnership between 16 countries in the Sub-Saharan Africa and 14 countries in the European Union aims to enhance research capacity of these developing countries while accelerating the development of health technologies.

Further studies could also examine the role of PPPs in increasing trial density in underserved regions. Understanding how these collaborations can effectively support the expansion of clinical research in LMICs while ensuring ethical standards and local benefits is essential. Additionally, research should explore the impact of these partnerships on the sustainability of research infrastructure and the long-term availability of new therapies in these regions.

This study has several limitations such as the potential bias in data sources, which result in underreporting of trials in certain countries or regions. The evolving coverage ratio of ClinicalTrials.gov adds another layer of uncertainty, as the completeness and representativeness of the data may vary over time. As the European Union Drug Regulating Authorities Clinical Trials Database (EudraCT), Japanese Primary Registries Network (JPRN), and Chinese Clinical Trial Registry (ChiCTR) were not designed to function as analytical tools, no application programming interface or web service is available to extract data from these registries. Clinical trial information can therefore only be accessed manually as plain text on these websites, significantly limiting the scope for comprehensive analyses. This limitation prompted us to rely exclusively on ClinicalTrials.gov for our analysis. On the contrary, the cross-sectional nature of the data does not allow for a causal interpretation of the factors influencing trial density. It is therefore difficult to determine whether and which variables have a direct impact on the distribution of clinical trials.

Additionally, important fields for search, such as condition and intervention, are not restricted to standardized ontologies, which complicates the consistency and reliability of the data. Notably, almost half of the conditions are not denoted by Medical Subject Headings (MeSH) terms, which are recommended for improving search accuracy and uniformity.^34^ These limitations highlight the importance of improving data reporting and standardization in future research efforts. Finally, to improve access to comprehensive clinical trial data worldwide, prioritizing data standardization and digitalization across regions is essential. The adoption of universal frameworks for clinical outcome measures, such as the Common Terminology Criteria for Adverse Events, performance status scales, and the TNM (tumor–node–metastasis) classification for staging, could help achieve consistent data collection, enhancing the comparability and reliability of trial outcomes across diverse settings.

## Conclusion

This study highlights significant shifts and persistent disparities in global oncology clinical research over the past 20 years. While the number of clinical trials has grown substantially, most of the research activity remains concentrated in HICs. Progress in the timely registration and ethical conduct of trials is encouraging, yet challenges persist in ensuring equitable access to trials and broad representation across diverse populations. To address these challenges and reduce the global cancer burden, ongoing investments in research infrastructure and a strong commitment to ethical standards are key, particularly in regions where the need for advanced cancer treatments is most pressing.
