# Supplementary material for: Globalization of clinical trials in oncology: a worldwide quantitative analysis
Source: ESMO Open. 2024 Dec 18;10(1):104086. doi: 10.1016/j.esmoop.2024.104086 (PMC11728923; doi:10.1016/j.esmoop.2024.104086)
Supplement: Supplementary Materials [file mmc1.pdf]

Table of content

Supplementary Table S1. Search strings. 2

Supplementary Table S2. First 10 countries with the highest trial density per million of habitants in 2024. 4

Supplementary Figure S1. Flowchart of included trials 5

Supplementary Figure S2. Absolute count of starting trials each year, according to their purpose. The solid line represents the correlation line between 2000 and 2021, and the dashed line the predicted values. 6

Supplementary Figure S3. Evolution of trial sites-year per country between 2005 and 2021 (%). 7

Supplementary Figure S4. Proportion of trials with industry or academic funding. 8

Supplementary Figure S5. Proportion of trials registered before first inclusion. 9

Supplementary Figure S6. Proportion of validation phase trials with survival criterion as primary outcome. 10

Supplementary Table S1. Search strings.

<table>
<tr><th>Platform or software used</th><th>Text pattern</th></tr>
<tr><td>ClinicalTrials.gov</td><td>cancer; Neoplasms; Tumors; Tumor; Neoplasm; malignancies; Cancers; Oncology; Malignancy; tumours; Tumour; Neoplasia; Malignant neoplasm; malignant tumors; Malignant tumor; Malignant Neoplasms; primary cancer; Neoplastic Disease; Cancer NOS; neoplasias; neoplastic syndrome; Primary Malignant Neoplasm; tumor NOS; Malignant tumour; Malignant neoplastic disease; malignant tumours; malignant neoplasm primary</td></tr>
<tr><td>R software version 4.4.1 (R Foundation for Statistical Computing, Vienna, Austria), package stringr version 1.5.0</td><td>Related to oncology (general):
Cancers* | Neoplas | Malignan | Oncol | Tumou*rs* | Lesions* | Nodules* | Metast

Related to tumor histologies:
Barrett | Mesotheliom | Thymom | Thymic | Thymect | N*SCLC | MPM | HCC | MBC | HSPC | HNSCC | SCC | m*HSPC | CRPC | CSPC | CRC | UCNT | N*MICB | Carcin | Sarcom | Chordoma | Ewing | Desmoid | GIST | Melano | Merkel | Medulloblastoma | Neuroblastoma | Nephroblastoma | Wilms | Adenom | Gliom | Glial | Glioblastoma | Astrocytoma | Meningioma | Schwannoma | Hemangioendothelioma | Hemangioma | Pseudomyxoma | Insulinom | Gastrinoma | Carcino | Cushing | Pheochromo | Seminoma | Teratoma | Choriocarcinoma | Germinoma | Ependymoma | ACP | Craniopharyngioma

Related to hematological malignancies:
Hemopath | Hematologic malign | Lymphom | Hodgkin | NHL | Leuka*em | Myelom | Amyloidos | Myelodysp | Myelofib | Waldenstrom | Waldenström | Sezary | DLBCL | MDS | CML | AML | ALCL | LAM | LLC | MPN

Related to systemic treatment:
CAR-T | Chemother | TKI | Tyrosine | Immunothe | Hormon | endocrine therapy | Neutropen | Lymphopen | Lymphedem | Extravasation | Thrombo | Neuropathy | Uricemia

Related to systemic treatments:
CAR-T | Chemother | TKI | Tyrosine | Immunothe | Hormon | endocrine therapy | Neutropen | Lymphopen | Lymphedem | Extravasation | Thrombo | Neuropathy | Uricemia

Related to radiotherapy:
radiation therapy | radiotherapy | proton | radiochemo | chemoradi | chemo-radi | brachy | plesio | IGBT | IGRT | conformal | 3DCRT | IMRT | SBRT | SRS | radiosurgery | cyberknife | fraction | grays | boost | irradiation | Hyperbar | Radionecro | Radiodermatitis | Radiation dermatitis | Mucositis</td></tr>
</table>

	Molecular biology:<br>4-1BB | 53BP | CD137 | Adenosine | ALK | APC | ARID1 | ATM | ATR | BAP1 | BARD1 | BCL | BCR | BRAF | BRCA | BRD | BTK | CALR | CD3 | CD5 | CD8 | CD19 | CD20 | CD22 | CD34 | CD47 | CD73 | CDKN2 | C-KIT | CSF1 | CTLA | CXCL | DLL3 | EGFR | ESR1 | EWSR | EZH2 | FGF | FOXP | HER*2 | HER*3 | IDH*1 | IL*6 | IL*13 | KEAP1 | KRAS | LAG*3 | MAGE*A4 | Mesothelin | MEK | MET | MDM2 | MGMT | MRE11 | MSI | MMR | MLH1 | MSH2 | MSH6 | PMS2 | NY*ESO | NECTIN | NETRIN | NKG2 | NRAS | LAG3 | OX40 | P16 | P53 | PALB2 | PARP | PDGF | PIC3 | PTEN | RAD51 | RET | ROS1 | PDL*1 | SMARC | STK11 | TERT | TIGIT | TIM*3 | TLR | TRK | TROP*2 | VEGF | VHL | VISTA | WNT | ICOS | CD78 | IDO | Indoleamine | indoleamine | NUT<br><br>Other terms:<br>Integrative | Pain | Opioid | Supportive | Palliative | Screening | Healthy volunteers | patient reported | PROM

Supplementary Table S2. First 10 countries with the highest trial density per million of habitants in 2024.

	Site	Number of trial sites-year			Density of trials (per million of habitants)		Relative evolution between 2000 and 2021 (%)	Population (millions)	Income group
		2005	2021	2024 (on June 13th)	2005	2021			
1	USA	62652	277658	251686	186.11	824.79	343.17	336.6	High income
2	Belgium	880	4938	4138	75.98	426.35	461.14	11.6	High income
3	Spain	1715	18140	15988	36.18	382.72	957.73	47.4	High income
4	France	3019	24728	22134	44.71	366.19	719.08	67.5	High income
5	Israel	282	3037	2927	31.94	343.98	976.95	8.8	High income
6	Denmark	237	1812	1594	40.58	310.22	664.56	5.8	High income
7	Australia	1332	7879	7321	51.64	305.43	491.52	25.8	High income
8	Switzerland	572	2451	2002	65.97	282.67	328.5	8.7	High income
9	Netherlands	691	4894	4468	39.56	280.19	608.25	17.5	High income
10	Italy	2327	16513	14044	39.2	278.18	609.63	59.4	High income

Abbreviations: USA, United States of America

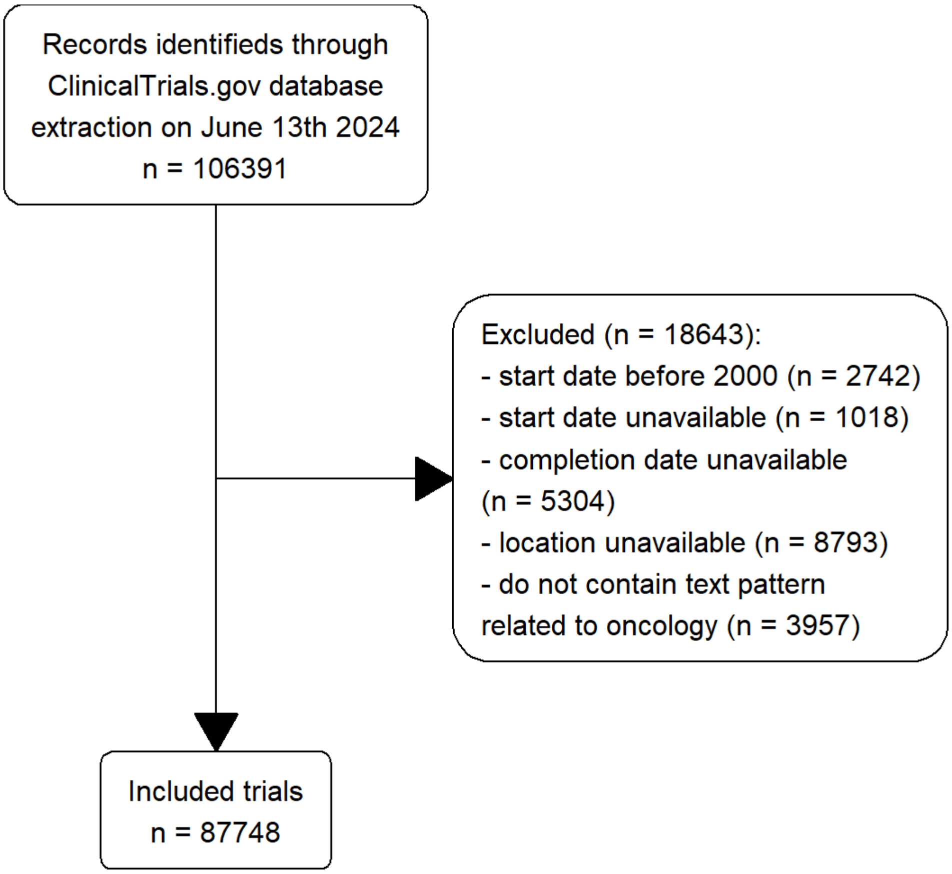

Supplementary Figure S1. Flowchart of included trials

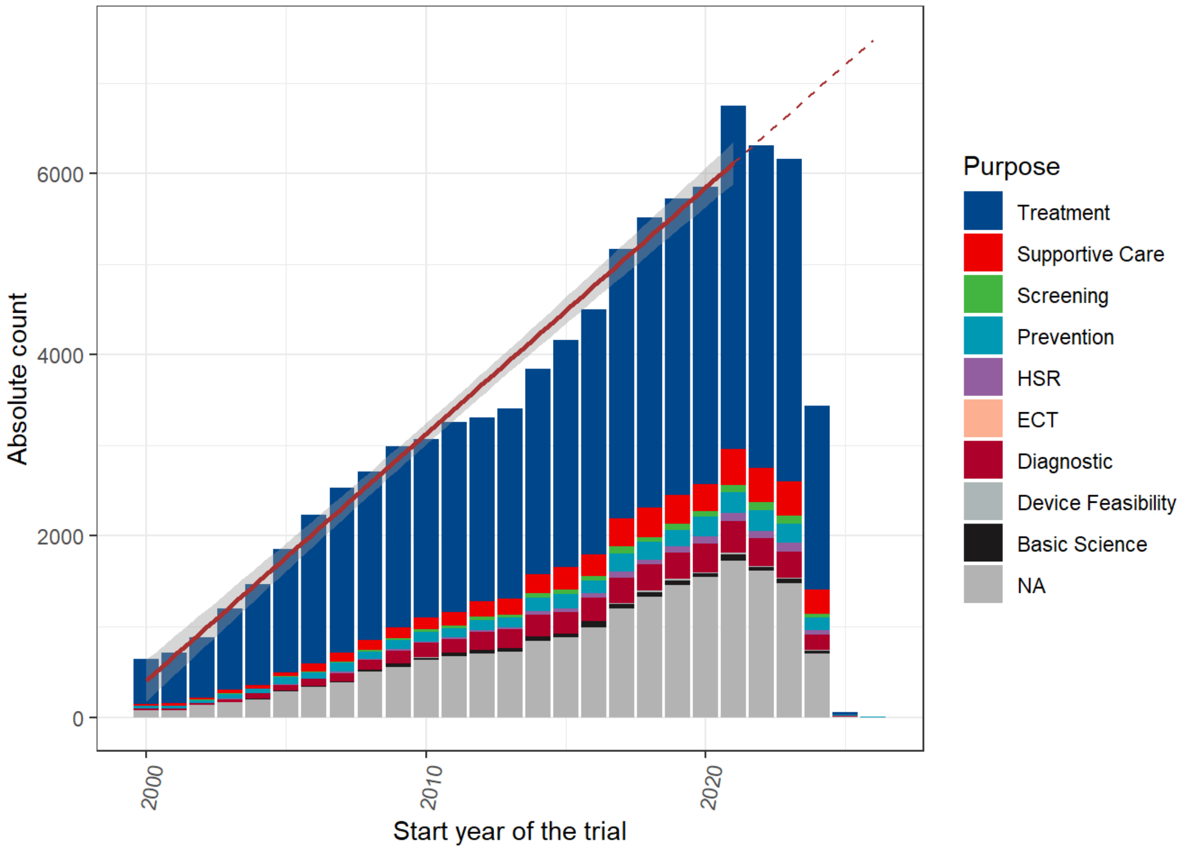

Supplementary Figure S2. Absolute count of starting trials each year, according to their purpose. The solid line represents the correlation line between 2000 and 2021, and the dashed line the predicted values.

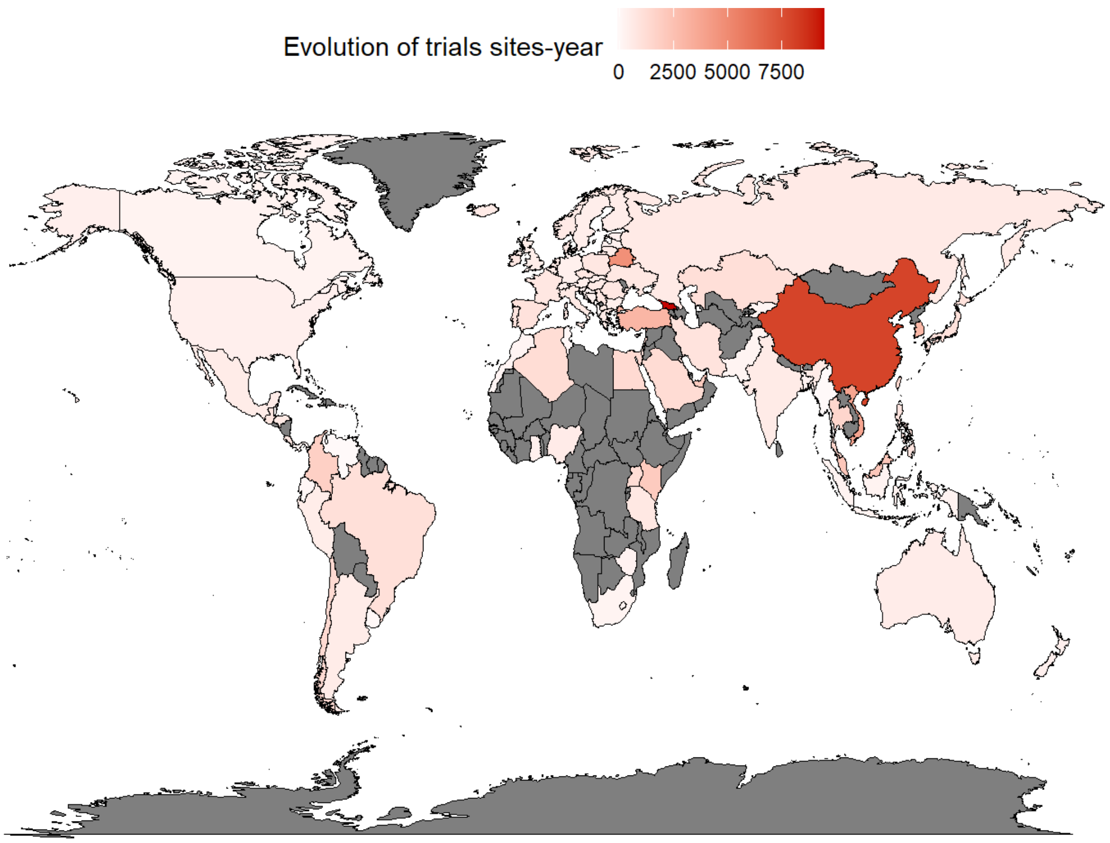

Supplementary Figure S3. Evolution of trial sites-year per country between 2005 and 2021 (%).

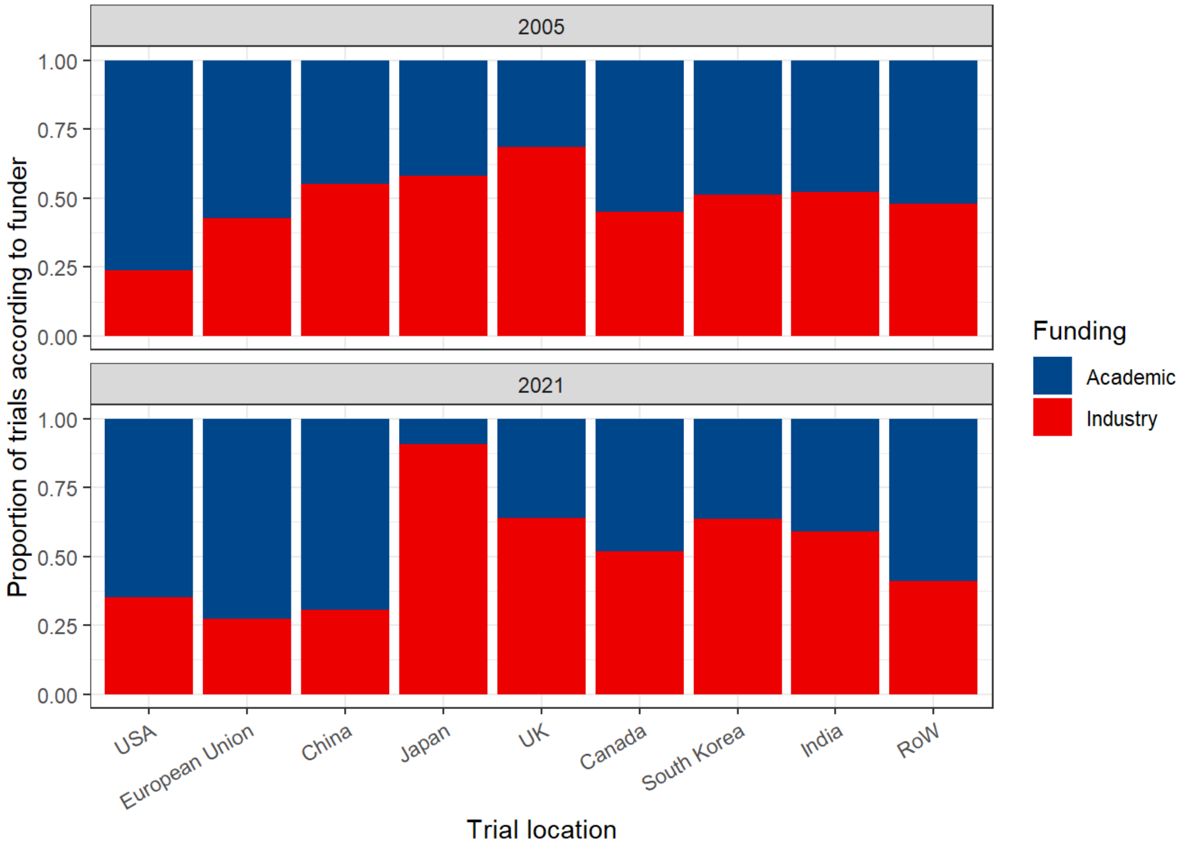

Supplementary Figure S4. Proportion of trials with industry or academic funding.

Abbreviations: USA, United States of America; UK, United Kingdom; RoW, rest of the world

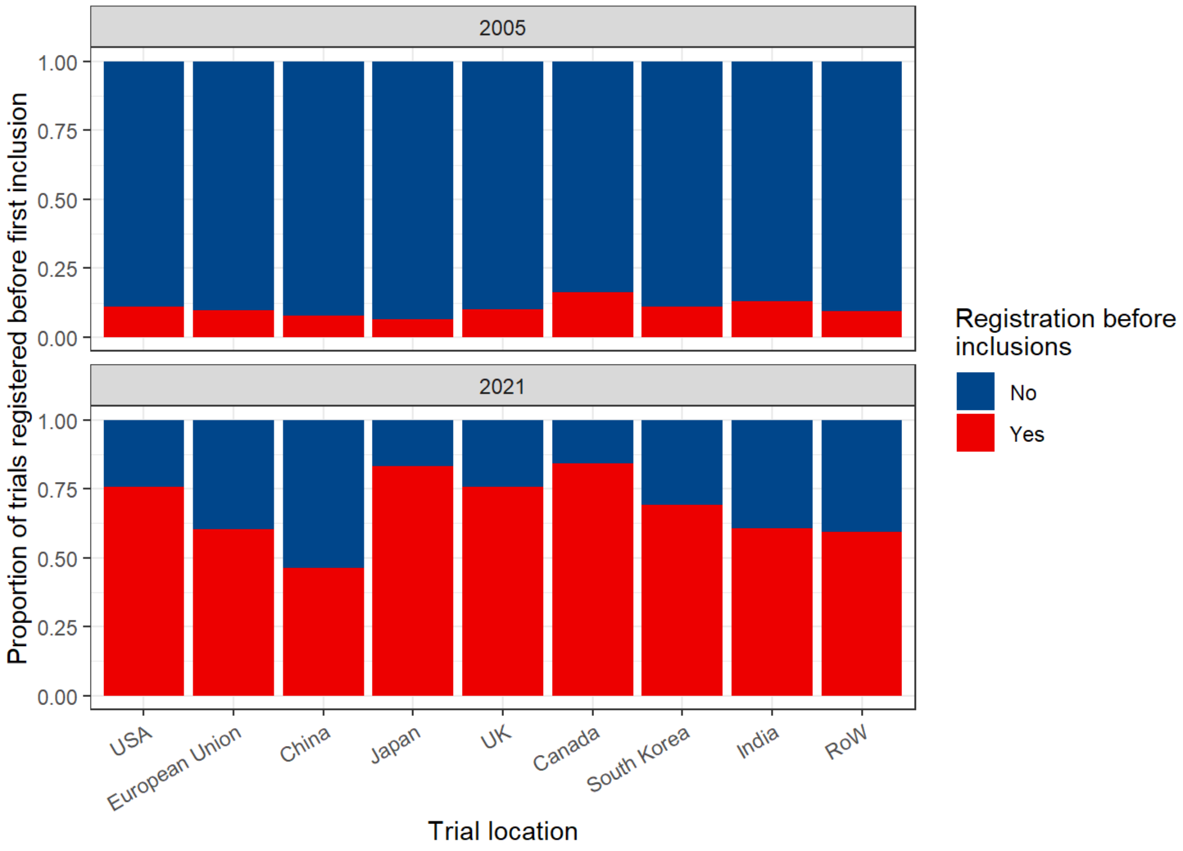

Supplementary Figure S5. Proportion of trials registered before first inclusion.

Abbreviations: USA, United States of America; UK, United Kingdom; RoW, rest of the world

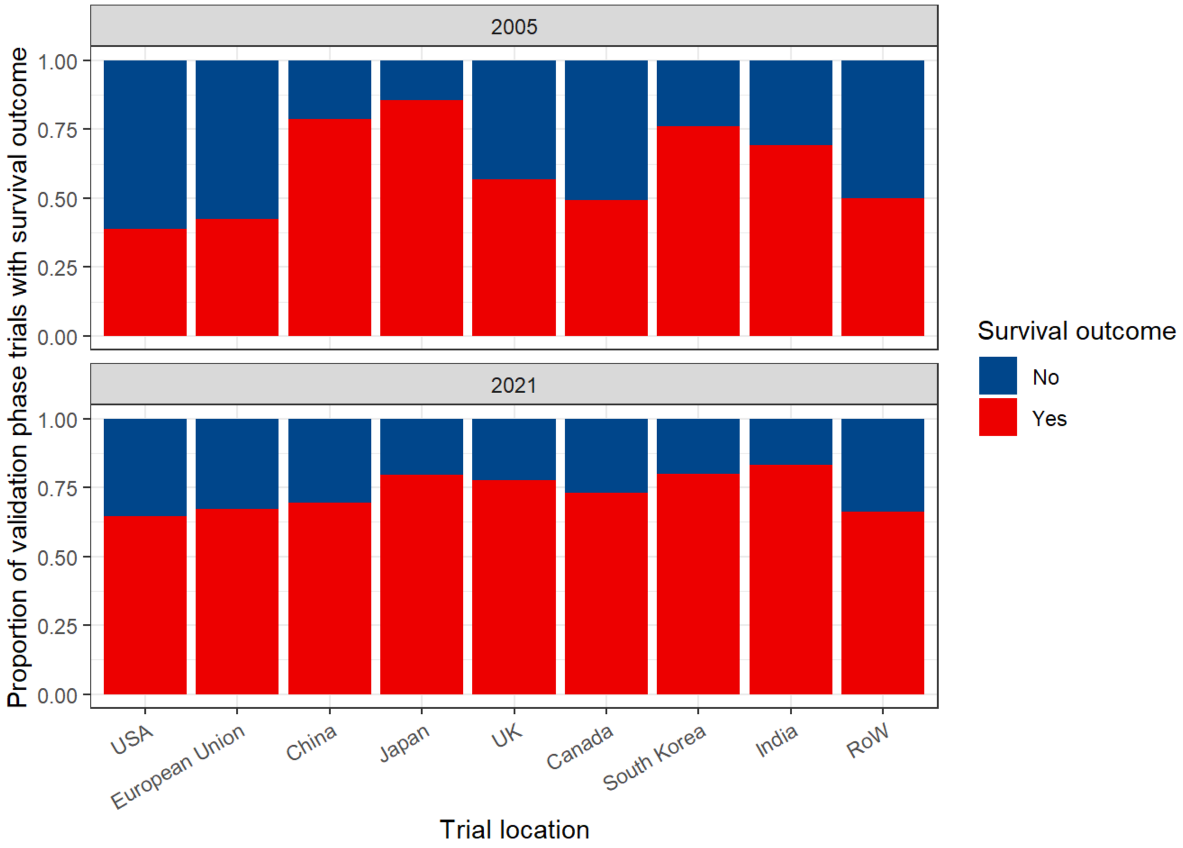

Supplementary Figure S6. Proportion of validation phase trials with survival criterion as primary outcome.

Abbreviations: USA, United States of America; UK, United Kingdom; RoW, rest of the world
